# Value of Prominent Flow Voids without Cord Edema in the Detection of Spinal Arteriovenous Fistulae

**DOI:** 10.1371/journal.pone.0099004

**Published:** 2014-06-06

**Authors:** Lea M. Alhilali, Arich R. Reynolds, Saeed Fakhran

**Affiliations:** Department of Radiology, University of Pittsburgh Medical Center, Pittsburgh, Pennsylvania, United States of America; University Medical Center (UMC) Utrecht, Netherlands

## Abstract

**Purpose:**

To determine the prevalence of spinal dural arteriovenous fistulae (SDAVF) in patients presenting with prominent vascular flow voids on imaging without other imaging findings suggestive of SDAVF.

**Methods:**

We retrospectively identified patients from January 1, 2005 to March 1, 2012 who underwent spinal angiography for suspected SDAVF with prominent vascular flow voids on prior imaging. We excluded patients with other major spinal pathology or other imaging findings of SDAVF including cord hyperintensity, enhancement, or expansion. We calculated the proportion of patients with positive findings for SDAVF on angiography and evaluated the prevalence of SDAVF for this finding alone and in correlation with clinical findings.

**Results:**

18 patients underwent spinal angiography for prominent flow voids on imaging without other spinal pathology or imaging findings of SDAVF. Three had a SDAVF detected on angiography. The prevalence of SDAVF in this population was low, only 17% (95% CI 6-39%). All of the patients with positive angiography findings had myelopathy, increasing the prevalence to 100% if the additional clinical finding of myelopathy was present.

**Conclusions:**

Prominent flow voids without other imaging findings suggestive of SDAVF is poorly predictive of the presence of a SDAVF, unless myelopathy is present clinically.

## Introduction

Although spinal dural arteriovenous fistulae (SDAVF) account for approximately 70% of all spinal vascular malformations, they still remain a relatively rare entity [1], [2]. The symptomatology associated with SDAVF is nonspecific and may mimic much more common pathologies including degenerative disc disease, radiculopathy, spinal stenosis and peripheral neuropathy [3]–[6]. Early detection is key, as prognosis depends on the duration of symptoms [1]. With its nonspecific clinical presentation, conventional imaging may be the first to suggest a SDAVF as the causative pathology. T2 hyperintensity within the cord has been shown to be 100% sensitive for detection of SDAVF[7], [8], but may be a later finding, as it indicates edema and hypoxia which are long-term sequelae resulting due to venous hypertension from the SDAVF [3].

Dilated intradural veins, seen as dilated flow voids on T2 weighted imaging, may be an earlier sign of venous hypertension and are occasionally seen before venous hypertension causes cord signal changes. Abnormal intradural vasculature without cord signal abnormality is usually seen in patients with milder disability, which is the ideal detection time-point, as greater initial disability is correlated with poorer outcome [9]. Recently, a case-series has reported that abnormal flow voids without cord signal changes have been used to detect essentially incidental SDAVFs, where all the SDAVFs detected with this isolated sign were asymptomatic [10]. However, there is overlap between the appearance of flow voids in SDAVF and the appearance of flow voids in normal patients [11], [12]. Additionally, prior studies evaluating the diagnostic value of flow voids in patients with SDAVF have included patients both with flow voids and abnormal cord signal [7], which when seen together may increase the specificity of the flow voids in diagnosing SDAVF, as compared to flow voids as an isolated finding. The purpose of our study was to determine the prevalence of SDAVF in patients with prominent flow voids as the only imaging finding suggestive of SDAVF on MR, using catheter angiography as the gold standard. We further sought to determine if there were any specific imaging or clinical findings that distinguished patients with prominent flow voids and positive angiographic findings from those with a negative angiogram.

## Materials and Methods

### Ethics Statement

Our institutional review board approved this study, with waiver of informed consent. All imaging and angiographic examinations included in this study were performed as standard of care and the results were retrospectively reviewed.

### Patient Selection and Image Acquisition

We searched our enterprise-wide electronic medical record, encompassing 20 academic and community hospitals, including a free-standing pediatric hospital, in an effort to identify patients who underwent spinal angiography for suspected SDAVF between January 1, 2005 to March 1, 2012. All spinal angiography reports were searched using the key-words “spinal dural arteriovenous fistula”, “SDAVF,” “spinal fistula,” “spinal dural AVF,” and “spinal dural AV fistula,”. Additionally, MR reports were searched with the keywords “dilated vasculature,” “dilated vessels,” “abnormal vasculature,” “abnormal vessels,” “prominent flow voids,” and “abnormal flow voids.” Patients were excluded if age was less 18 years, prior cross-sectional imaging or spinal angiography was not available for review (8 patients), if there was evidence of spinal pathology other than a SDAVF on imaging excluding minor degenerative findings based upon review by a fellowship-trained neuroradiologist both with certificates of added qualification (2 patients with hypervascular metastases), or angiography was performed more than 30 days after cross-sectional imaging (1 patient). Pediatric patients were not included in this study, as the pathology of spinal vascular malformations in children is significantly different than in the adult population, consisting of vascular malformations both with and without a nidus and are often associated with syndromes such as Weber-Osler-Rendu, Parkes Weber, Klippel-Trenaunay, and Cobb syndrome [13]. Additionally patients were excluded if there were other imaging findings of SDAVF including cord hyperintensity, enhancement, or expansion based upon review by a fellowship-trained CAQ certified neuroradiologist (30 patients). At our institution, MR examinations of the spine are interpreted by fellowship-trained neuroradiologists. Although the current study was a retrospective review, the determination of prominent vasculature for this study utilized the prospective interpretation by the initial interpreting neuroradiologist. Demographic data collected included age and sex. Clinical data collected included presenting symptoms, imaging and angiography results, and post-imaging clinical management.

MR examinations were performed on a 1.5T system (GE Healthcare, Milwaukee, Wisconsin) with neutral positioning using a standard spine coil. Sagittal sequences were obtained with 24 cm FOV and 256×192 matrix for the cervical and lumbar spines and a 32 cm FOV and 512×224 matrix for the thoracic spine as follows: sagittal spin echo T1-weighted (TR, 500 msec; TE, min; section thickness, 3 mm; number of acquisitions, 3), sagittal fast spin echo T2-weighted fat saturation (TR, 3500 msec; TE, 84-102 msec; section thickness, 3 mm; number of acquisitions, 3). Axial images were obtained with 22 cm FOV and 256×192 matrix for the cervical and lumbar spine and 24 cm FOV and 256×224 matrix for the lumbar spine as follows: axial spine echo T1-weighted (TR, 500 msec; TE, min; section thickness, 3 mm; number of acquisitions, 2), fast spin echo T2-weighted images (TR, 3500; TE 102–120 msec; section thickness, 3mm; number of acquisitions, 2). Additional axial 3D gradient echo images (TR, 35 msec; TE, 13 msec; flip angle 5; section thickness, 2 mm; number of acquisitions, 1) were obtained with a 22 cm FOV and 256×192 matrix in the cervical spine. If contrast enhanced T1-weighted images were obtained, post-contrast imaging was performed with 0.1 mmol/kg gadolinium-based contrast material (Multihance: Bracco, Milan, Italy) using typical T1-weighted parameters as described above. At our institution, post contrast imaging of the spine is performed immediately (<1 minute) after contrast administration.

Digital subtraction angiograms were performed within 30 days of the MR examinations. At our institution, the standard protocol is to inject all segmental arteries from the level of the vertebral arteries to the level of the internal iliac arteries. Segmental arteries were injected with approximately 3 cc of iohexol (Omnipaque, GE Healthcare, Milwaukee, Wisconsin; 300 mg of iodine/mL). Images were obtained at a rate of 2–4 frames/sec with a 1024×1024 matrix. Identification of the anterior spinal artery occurred in all cases. All segmental arteries were selectively catheterized and injected in all patients.

### Image Analysis

All MR examinations that were prospectively interpreted by a fellowship-trained neuroradiologist as having prominent vasculature were re-reviewed in consensus by 2 additional fellowship-trained, neuroradiologists with certificates of additional qualification and more than 2 years of experience. Images were re-evaluated for findings that have previously been found to be suggestive of abnormal vasculature on conventional imaging [11], [14], [15], [16]: (1) flow voids spanning more than 3 vertebral body levels; (2) serpentine appearance of the vessels; (3) largest size >2 mm; (4) detection of more than two vessels. Inter-rater agreement for these imaging findings was determined utilizing Cohen's kappa: two fellowship-trained neuroradiologists were separately asked to determine the presence or absence of each of the four imaging findings indicative of abnormal vasculature for each study, blinded to both the clinical and angiographic findings as well as the other's interpretation. Kappa results were interpreted according to Altman (1.0 =  perfect agreement, 0.80–0.99 =  very good agreement, 0.60–0.79 =  good agreement, 0.40–0.59 =  moderate agreement, 0.20–0.39 =  fair agreement, and less than 0.20 =  poor agreement) [17]. Studies were then scored in consensus by the two neuroradiologists using these findings and given 1 point for each finding, for a maximum total score of 4. Additonally, the number of vertebral body levels spanned by any abnormal vasculature was recorded.

### Data Analysis

Comparison of proportions in the demographic and imaging analysis data was performed using Fisher's exact test. Comparison of continuous variables in the demographic and imaging analysis data was performed with a two-tailed unpaired t-test. Confidence intervals for the prevalence were calculated using the interval estimation for a binomial proportion [18]. Comparison of the number of vertebral body levels spanned by the abnormal vasculature between patients with and without SDAVF was performed with a two-tailed unpaired t-test. P values were two-tailed and p values less than 0.05 were considered significant.

## Results

18 patients (8 male, 10 female; mean age 49 years, range 2–88) underwent spinal angiography for suspected SDAVF with prominent flow voids as the only relevant imaging finding suggestive of SDAVF. The majority of patients presented with back pain (12 patients, 67%); three patients presented with myelopathy (17%).

Inter-rater agreement for the finding of flow voids spanning more than 3 vertebral body levels was perfect (kappa  = 1.0, 95% CI 1.00–1.00), while agreement was very good for determining serpentine appearance of the vessels (kappa  = 0.83, 95% CI 0.65-1.00) or the detection of more than two vessels (kappa  = 0.89, 95% CI 0.74-1.00). Inter-rater agreement was good for the finding of largest size >2 mm (0.67, 95% CI 0.43–0.99). The average score for imaging findings suggestive of abnormal vasculature in these patients was 2.4 (median 2, range 1–4). Ten patients had abnormal vessels spanning more than 3 vertebral levels (56%); eleven had serpentine vessels (61%); only six had vessels greater than 2 mm in size (33%); and the large majority had more than 2 vessels detected (16 patients, 89%). Demographic and clinical characteristics are summarized in Table 1.

**Table 1:** Demographic and clinical characteristics of patients undergoing myelography with prominent flow voids as the only imaging finding suggestive of SDAVF.

Patient No.	Age	Sex	Presenting Symptom	Angiography Findings
1	39	F	Myelopathy^1^	SDVAF from right T6 and T7 radicular arteries
2	63	F	Post-laminectomy syndrome	Negative
3	73	M	Thoracolumbar pain	Negative
4	26	F	Back pain	Negative
5	27	F	Bilateral lower extremity pain	Negative
6	43	M	Back pain	Negative
7	20	F	Chiari Malformation	Negative
8	63	F	Back pain	Negative
9	53	F	Bilateral lower extremityweakness, myelopathy^2^	SDVAF arising from the left L1 radicular artery
10	51	F	Lower extremity pain	Negative
11	60	M	Back pain	Negative
12	59	M	Back pain	Negative
13	45	M	Back pain	Negative
14	42	F	Back pain	Negative
15	64	M	Bilateral lower extremity pain	Negative
16	60	M	Back pain	Negative
17	88	F	Back pain	Negative
18	47	M	Back pain and myelopathy^3^	SDVAF arising from the right T12 radicular artery

1 Myelopathic symptoms of progressive loss of strength and sensation in the lower extremities.

2 Myelopathic symptoms of urinary retention and constipation.

3 Myelopathic symptoms of abnormal sensation in the lower extremities.

Three patients had a spinal angiogram positive for a SDAVF (1 male, 2 females; mean age 46.3, range 39–53). All three patients had type 1 SDAVF according to the Ansler and Spetlzer criteria [19] and were treated with embolization without findings of residual SDAVF on subsequent catheter angiography.

**Figure 1:**
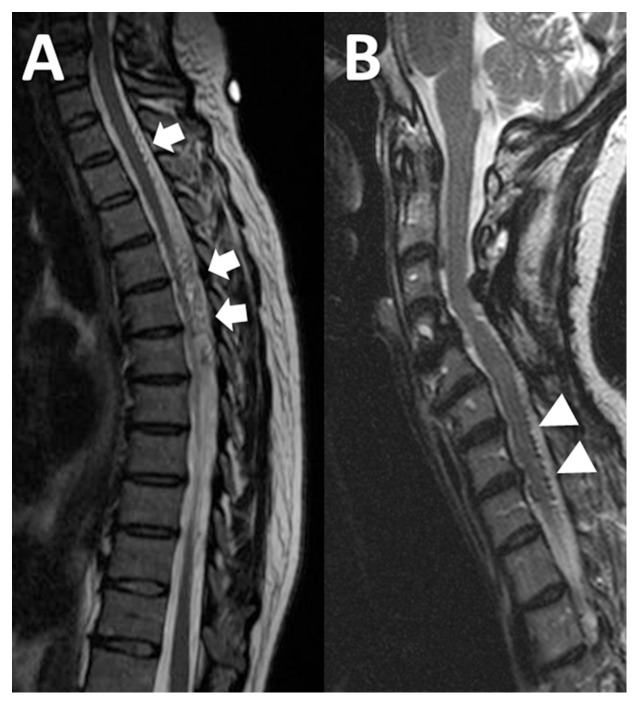
Abnormal flow voids without cord signal are seen in both patients with and without SDAVF. Sagittal T2-weighted images show prominent flow voids in (a) a patient with a positive spinal angiogram (arrows) and (b) a patient with negative angiography results (triangular arrowheads).

The prevalence of SDAVF in our population with only prominent flow voids was 17% (95% CI 6-39%). Figure 1 shows a comparison of images between three patients with prominent flow voids on MR, one with a positive angiogram and two without findings on angiography.

Of the 15 patients with negative angiography results, seven continued to be followed for non-specific back pain by a primary care physician. Presenting symptoms were ascribed to intracranial pathology without spinal involvement in three patients (1 melanoma brain metastases, 1 multiple sclerosis, and 1 normal pressure hydrocephalus). In two patients, the presenting symptom of pain was felt to be related to diabetic neuropathy and in one it was attributed to restless legs syndrome. One patient underwent spinal decompression for epidural lipomatosis as the presumed etiology of the presenting symptoms. In the remaining patient, the presenting pain was felt to be related to a prior cervical cord injury that was not imaged and the patient was treated with an intrathecal catheter.

There was no significant difference in the demographic characteristics between patients with and without positive angiography findings. Similarly, no significant difference was seen in the scores for findings suggestive of abnormal vasculature or in the presence of vessels spanning more than 3 vertebral body levels, serpentine vessels, vessels larger than 2 mm, or more than 2 detected vessels. The average extent of the abnormal vasculature between patients with and without SDAVF trended towards, but did not reach statistical significance (mean number of vertebral body levels spanned by abnormal vasculature  = 6.0 and 3.7, range 1–7 and 4–8, respectively, p = 0.07).

Of note, all three patients with positive angiography findings had myelopathy present clinically and no patients with a negative angiogram had clinical myelopathy (p = 0.001). No patients with a score less than 3 had a positive angiogram. Comparison of the demographic and imaging data between the two groups is summarized in Table 2.

**Table 2:** Comparison of demographic and imaging data for patients with and without positive findings on angiography.

	Patients with Negative Angiogram	Patients with Positive Angiogram	p value
**Number**	15	3	N/A
**Number male** (%)	7 (47%)	1 (33%)	0.6
**Age** (yrs, mean, range)	49.7 (20–88)	46.3 (39–53)	0.61
**Score for findings suggesting abnormal vessels** (mean, range)	2.2 (1–4)	3.3 (3–4)	0.2
**Vessels spanning** more than 3 levels (number, percent)	7 (47%)	3 (100%)	0.22
**Serpentine vessels** (number, percent)	8 (53%)	2 (66%)	1
**Vessel >2 mm size** (number, percent)	5 (33%)	2 (66%)	0.53
**Detection of more** **than 2 vessels** (number, percent)	13 (87%)	3 (100%)	1
**Presence of myelopathy** (number, percent)	0 (0%)	3 (100%)	0.001

## Discussion

Prominent flow voids without other imaging findings of SDAVF were found to be poorly predictive of the presence of a SDAVF, unless myelopathy was present clinically. No patients with less than three of the four imaging findings previously found to be suggestive of abnormal vasculature had a positive angiogram.

Previous studies evaluating the value of flow voids in the diagnosis of SDAVF have included patients with both flow voids and abnormal cord signal [3], [7], [10], [20], therefore obscuring the value of this finding in isolation. A recent study has indicated the importance of suggesting the diagnosis of SDAVF in the setting of prominent flow voids, even in the absence of other imaging findings suggestive of SDAVF or myelopathic symptoms, given the possibility of detecting a SDAVF before it becomes symptomatic [10]. However, this study only retrospectively evaluated patients with positive angiographic findings and did not evaluate the prevalence of prominent flow voids in patients with negative angiographic studies. Thus, radiologists have been left with the impression that they may potentially miss a curable spinal myelopathy if SDAVF is not routinely mentioned in the setting of prominent flow voids. However, our results would suggest such an approach would result in a very high number of false positive studies. False positives are significant in the workup of a SDAVF, as they can result in catheter angiography, which has significant risks, even in the hands of experienced practitioners, as well as the potential for nephrotoxicity associated with use of iodinated contrast [3].

Our study is the first to evaluate for SDAVF in all patients with prominent vasculature as the only finding of SDAVF on conventional imaging, with catheter angiogram for definitive diagnosis. Previous studies examining prominent flow voids without abnormal cord signal have only evaluated its value in populations with known SDAVFs [10], [21]. No study has yet evaluated this finding in the general population to determine its associated false positive rate for diagnosing a SDAVF. In contradistinction to the prior case series [10], we did not find any patients with a SDAVF with only prominent flow voids on imaging and without myelopathy on clinical examination. The presence of myelopathy without abnormal cord signal in our patients may reflect venous hypertension high enough to cause decreased cord perfusion and myelopathy, but not substantial or long-standing enough to cause edema and abnormal cord signal [3]. This correlates with the known lack of association between cord signal and symptoms after treatment of the SDAVF [22]. Lack of both abnormal cord signal and myelopathy suggests the prominent vasculature does not reflect venous hypertension but rather anatomic variation, except perhaps in rare, reportable cases [10]. Therefore, in patients with prominent vascular flow voids but no abnormal cord signal, given the relative rarity of SDVAF in this population, correlation for a history of myelopathy should be undertaken.

None of the patients with less than three of the four imaging findings previously found to be suggestive of abnormal vasculature had a SDAVF. The presence of any one of the four findings may result in a subjective appearance of prominent vasculature on imaging. However, based on our findings, we would advise that determination of abnormal vasculature should not be based on a simple overall impression, which may be influenced by only one or two of the flow void findings. Rather, if there is a suspicion that the vasculature may be abnormal, a dedicated evaluation for the known four imaging findings of flow voids in SDAVF should be undertaken as well as a search of the medical record, or discussion with the referring physician, for a history of myelopathy. Additionally, a further workup to assess for subtle myelopathic findings on physical examination or subtle imaging findings of a SDAVF, including possible MR angiography, may be helpful [23]. Furthermore, a higher number of vertebral body levels spanned by the abnormal vasculature showed a trend towards significance, and has previously been correlated with the degree of myelopathy in patients with SDAVF [24]. Larger studies may be helpful to confirm this finding as a way to determine truly abnormal vasculature.

The principal limitation to this study is that our institution does not routinely perform spinal MR angiograms (spinal MRA). While, spinal MRA has emerged as a useful, noninvasive, tool in which to localize arterial feeders and venous drainage patterns in patients with high clinical imaging suspicion for, and corroborative imaging findings strongly suggestive of, SDAVF [25], [26], it has not been shown to improve sensitivity or specificity in detection of SDAVF in patients with equivocal conventional MR findings, such as lack of abnormal cord signal [11]. As a result, our spinal angiographers, believe that spinal MRA, in patients with such equivocal MR findings, to be of insufficient negative predictive value to preclude spinal angiography, and hence do not routinely order them in this subset of patients.

Additional limitations of this study include its retrospective nature and small sample size. However, SDAVF itself is a rare disease, and prominent flow voids and lack of abnormal T2 signal is an even rarer imaging presentation of the disease [9], [25], [26]. In selecting for patients only with a definitive angiographic study, we further decreased our potential patient population, as it rare to undertake invasive angiography with only a single imaging finding. However, we feel that this further strengthens our finding, as the patients included in our study underwent angiography either because the radiologist felt very strongly about the flow void appearance or there was strong clinical concern. The poor correlation between flow voids and SDAVF in this population with either high imaging confidence or clinical suspicion further supports that prominent flow voids without cord signal abnormality should not be considered abnormal on a routine basis.

## Conclusion

In conclusion, the prevalence of SDAVF in patients with prominent flow voids as the only imaging finding suggestive of SDAVF is low, particularly in patients without myelopathy and in those with less than three imaging findings to suggest abnormal vasculature.
